# Perimenopause and emergence of an Alzheimer’s bioenergetic phenotype in brain and periphery

**DOI:** 10.1371/journal.pone.0185926

**Published:** 2017-10-10

**Authors:** Lisa Mosconi, Valentina Berti, Crystal Guyara-Quinn, Pauline McHugh, Gabriella Petrongolo, Ricardo S. Osorio, Christopher Connaughty, Alberto Pupi, Shankar Vallabhajosula, Richard S. Isaacson, Mony J. de Leon, Russell H. Swerdlow, Roberta Diaz Brinton

**Affiliations:** 1 Department of Neurology, Weill Cornell Medical College, New York, NY, United States of America; 2 Department of Psychiatry, New York University School of Medicine, New York, NY, United States of America; 3 Department of Clinical Pathophysiology, Nuclear Medicine Unit, University of Florence, Florence, Italy; 4 Department of Radiology, Weill Cornell Medical College, New York, NY, United States of America; 5 Department of Neurology, University of Kansas School of Medicine, Kansas City, United States of America; 6 Departments of Pharmacology and Neurology, University of Arizona College of Medicine, Tucson, AZ, United States of America; Banner Alzheimer's Institute, UNITED STATES

## Abstract

After advanced age, female sex is the major risk factor for Alzheimer’s disease (AD). The biological mechanisms underlying the increased AD risk in women remain largely undetermined. Preclinical studies identified the perimenopause to menopause transition, a neuroendocrine transition state unique to the female, as a sex-specific risk factor for AD. In animals, estrogenic regulation of cerebral glucose metabolism (CMRglc) falters during perimenopause. This is evident in glucose hypometabolism and decline in mitochondrial efficiency which is sustained thereafter. This study bridges basic to clinical science to characterize brain bioenergetics in a cohort of forty-three, 40–60 year-old clinically and cognitively normal women at different endocrine transition stages including premenopause (controls, CNT, n = 15), perimenopause (PERI, n = 14) and postmenopause (MENO, n = 14). All participants received clinical, laboratory and neuropsychological examinations, ^18^F-fluoro-deoxyglucose (FDG)-Positron Emission Tomography (PET) FDG-PET scans to estimate CMRglc, and platelet mitochondrial cytochrome oxidase (COX) activity measures. Statistical parametric mapping and multiple regression models were used to examine clinical, CMRglc and COX data across groups. As expected, the MENO group was older than PERI and controls. Groups were otherwise comparable for clinical measures and distribution of APOE4 genotype. Both MENO and PERI groups exhibited reduced CMRglc in AD-vulnerable regions which was correlated with decline in mitochondrial COX activity compared to CNT (p’s<0.001). A gradient in biomarker abnormalities was most pronounced in MENO, intermediate in PERI, and lowest in CNT (p<0.001). Biomarkers correlated with immediate and delayed memory scores (Pearson’s 0.26≤r≤0.32, p≤0.05). These findings validate earlier preclinical findings and indicate emergence of bioenergetic deficits in perimenopausal and postmenopausal women, suggesting that the optimal window of opportunity for therapeutic intervention in women is early in the endocrine aging process.

## Introduction

After advanced age, female sex is the major risk factor for developing late-onset Alzheimer’s disease (AD) [1]. While AD is not unique to the female, women constitute the majority of people with the disease, accounting for two-thirds of the 5.5 million Americans living with AD dementia in 2017 [2]. AD risk is greater in women even after accounting for their greater longevity relative to men [3].

Despite well-established vulnerability, the biological mechanisms underlying the increased risk of AD in women remain largely unknown. However, preclinical evidence implicates a shift in the bioenergetic system of the brain during the *perimenopause to menopause transition* which could serve as an early initiating mechanism for increased AD risk in the female brain [4].

The perimenopause to menopause transition is a midlife neuroendocrine transition state unique to the female that occurs on the background of an aging biology [4]. While the outcome of this transition is reproductive senescence, the related symptoms are largely neurological in nature. These include disruption of estrogen-regulated systems such as thermoregulation, sleep, circadian rhythms and sensory processing, as well as depression and impairment in multiple cognitive domains [4].

Chronologically, age of menopause maps onto the time course for initiation of the *prodromal phase* of AD, which typically begins 15–20 years before clinical symptoms emerge [5]. Menopausal changes therefore coincide with the timespan between average age of menopause, in the mid-50s, and average age of AD diagnosis, in the mid-seventies.

From a mechanistic perspective, estrogen dysregulation during perimenopause significantly affects brain bioenergetics [4]. The brain is dependent upon glucose as the principal metabolic fuel to generate ATP–a system that is partially regulated by estrogen. During perimenopause, estrogenic regulation of cerebral glucose metabolism (CMRglc) falters, inducing a hypometabolic state which is accompanied by deposition of amyloid-beta (Aβ, a hallmark of AD pathology), decreased mitochondrial function, and decline in synaptic plasticity [6–8].

Synaptic transmission consumes 75% of ATP generated in brain with cognitive function accounting for a large proportion of the required ATP [9]. Estrogen promotes aerobic glycolysis coupled to mitochondrial oxidative phosphorylation for generation of ATP [10]. Preclinical evidence indicated that the loss of brain estrogen, either by surgical removal of the ovaries or through natural endocrine aging, induces decline in glucose metabolism and mitochondrial function including cytochrome oxidase activity (COX, Complex IV of mitochondria electron transport chain, ETC) and ATP generation (for a recent review, see [10]).

Based on these findings as well as substantial evidence for altered brain bioenergetic early in the course of AD [11], we investigated brain CMRglc and mitochondrial COX activity, a rate limiting step in ATP production, in women across endocrine aging states.

Positron emission tomography (PET) studies with ^18^F-fluorodeoxy-2-glucose (FDG) as the tracer has consistently documented that reduction in brain CMRglc develop years, if not decades, prior to onset of clinical symptoms [12–16], correlate with AD progression [17], and are more severe in women than in men [18]. Glucose dysmetabolism in AD occurs in conjunction with mitochondrial dysfunction, particularly reduced COX activity in brain tissue, fibroblasts and platelets in humans, and is extensively documented in preclinical models of prodromal AD [11, 19–21].

Herein, we present a translational neuroimaging study that bridges basic to clinical science to characterize changes in ^18^F-FDG PET CMRglc and mitochondrial COX activity in a cohort of clinically and cognitively normal women at different endocrine transition stages (premenopause vs. perimenopause vs. menopause).

## Methods

### Participants

Among a larger pool of clinically and cognitively normal individuals participating in brain imaging studies at New York University (NYU) School of Medicine/Weill Cornell Medical College, this study focused on a sub-set of 62 female participants of age 40–60 years who completed clinical, labs, neuropsychological examinations, FDG-PET scans and a blood draw to measure mitochondrial COX activity between 2010–2015. Subjects were derived from multiple community sources, including individuals interested in research participation, family members and caregivers of impaired patients.

The study protocol has been previously published [15, 22]. Briefly, all subjects underwent thorough physical examinations with detailed medical histories. Individuals with medical conditions or history of conditions that may affect brain structure or function (e.g. stroke, unmanaged diabetes, depression, head trauma, any neurodegenerative diseases, hydrocephalus, intracranial mass, and infarcts on MRI), and those taking psychoactive medications were excluded. In order to be included in this study, subjects had to be 40–60 years of age, with education≥12 y, Clinical Dementia Rating (CDR) = 0, Global Deterioration Scale (GDS)≤2, Mini Mental State Examination (MMSE)≥27, Hamilton depression scale<16, Modified Hachinski Ischemia Scale<4 and normal cognitive test performance for age and education [15, 22].

The neuropsychological battery of tests was constructed as previously described [23]. Four cognitive domains were assessed from the following tests: memory (Immediate and delayed recall of a paragraph [PARI, PARD], and immediate and delayed recall of paired associates [PRDI, PRDD]), executive function (Wechsler Adult Intelligence Scale Digit Symbol Substitution [DSST]), language (Object Naming Test [ONT] and WAIS vocabulary), and visuospatial performance (Block Design test [DESN]).

DNA was obtained from venous blood samples to determine *APOE* genotypes using standard quantitative polymerase chain reaction (PCR) [15, 22]. Individuals with one or two APOE ε4 alleles were categorized as APOE ε4 carriers (APOE4+) and compared to non-carriers (APOE4-).

### Standard protocols approval, registration and patient consents

This study received approval from NYU School of Medicine and Weill Cornell Medical College institutional review boards. Written informed consent was obtained from all patients participating in the study.

### Determination of menopausal status

Only participants with detailed medical records of menopausal status were included in this study. Determination of menopausal status was based on clinical judgment, medical records and detection of cluster symptoms according to the Stages of Reproductive Aging Workshop (STRAW) criteria [24]. Premenopausal women have regular menstrual cycles that are <7 days variability in cycle length. Perimenopausal women have irregular menstrual cycles, with variation in cycle length >7 days. Postmenopausal women have had no menstrual cycles for ≥12 months. Detection of cluster symptoms was based on presence of sweatiness/hot flashes, mood swings, insomnia, change in appetite, loss of libido, cognitive problems/concentration and short-term memory complaints [24]. Based on these assessments, participants were classified into 3 groups: asymptomatic perimenopausal women by age (e.g., premenopausal controls, CNT); symptomatic perimenopausal (PERI); and postmenopausal women (MENO).

### COX analysis

After an overnight fast, 50 ml of blood were collected in tubes containing acid-citrate-dextrose. Blood samples were obtained at NYU and sent overnight to the Mitochondrial Genomics and Metabolism Core of the University of Kansas Alzheimer’s Disease Center. Upon receipt, platelets were isolated by centrifugation and enriched mitochondrial fractions were prepared using previously described methods [25]. The protein concentrations of the enriched mitochondrial fractions were measured using a DC protein assay kit (Bio Rad, Hercules, CA).

Cytochrome *c* oxidase Vmax activity (COX, Complex IV, sec^-1^/mg) was determined as a pseudo first order-rate constant by measuring the oxidation of reduced cytochrome *c* at 550 nm. In addition to referencing COX Vmax activity to total protein, to correct for potential inter-sample differences in mitochondrial mass the COX activity for each sample was also referenced to its corresponding citrate synthase (CS) activity. CS activity is reportedly comparable between NL and AD patients, with variations as little as 0.5–2%, and does not show age effects [26, 27]. CS Vmax activity (nmol/min/mg) was determined by spectrophotometrically following the formation of 5-thio-2-nitrobenzoate (412 nm) following the addition of 100 μM oxaloacetate at 30°C.

### Brain FDG-PET imaging

All subjects received FDG-PET scans at Weill Cornell Medical College following standardized procedures [15, 28]. Briefly, after an overnight fast, subjects were positioned in the scanner 35 minutes post injection of 5 mCi of ^18^F-FDG, and scanned for 20 min in 3D-mode on an LS Discovery or BioGraph PET/CT scanner. All images were corrected for attenuation, scatter and decay, and smoothed for uniform resolution [29].

For each subject, summed PET images corresponding to 40–60 min of FDG data were coregistered to the corresponding MRI using the Normalized Mutual Information routine of Statistical Parametric Mapping’12 (SPM’12), spatially normalized to a standard MRI template using subject-specific transformation matrixes obtained from MRI, and smoothed with a 10mm FWHM filter [30]. FDG uptake in the pons was used to normalize for inter-subject variability [31].

### Statistical analysis

SPSS v.22 (SPSS Inc.) and SPM’12 were used for data analysis.

Differences in clinical, demographical, and COX measures across groups were examined with χ^2^ tests, and the General Linear model (GLM) with post-hoc Tukey tests.

The total-protein COX Vmax was examined as absolute values and after adjustment for CS, which was examined both as a covariate and as a denominator (COX/CS).

For SPM PET analysis, a full factorial model with post-hoc *t*-contrasts was used to test for regional differences in FDG measures between groups, accounting for pons metabolism. Linear regressions were used to test for voxel-wise associations between COX activity and CMRglc measures across all subjects and by clinical group. Age, education, and APOE genotype were examined as covariates.

As we had specific *a priori* hypotheses on which brain regions would show possible FDG-PET effects, results were examined at p<0.001 after small-volume correction (SVC) within the search volume defined by a masking image created from a set of predefined bilateral AD-related regions of interest including posterior cingulate cortex, precuneus, parietal, temporal and frontal cortex [15, 22, 32]. The gray matter threshold was set at 0.8 and only clusters exceeding an extent threshold of 30 voxels were considered significant [32].

Anatomical location of brain regions showing significant effects was described using Talairach and Tournoux coordinates. CMRglc measures were extracted from clusters of voxels showing significant effects for further analyses.

Linear regressions and Pearson’s *r* determination coefficients were used to evaluate associations between CMRglc, COX, clinical and neuropsychological measures. An interaction term was included in the model to test for slope differences across groups.

Stepwise forward logistic regressions and ROC curves were used to examine COX and CMRglc as predictors of clinical group, and to calculate associated relative risk (RR) and 95% confidence intervals (C.I.). Results were considered significant at p<0.05.

## Results

### Subjects

Of the sixty-two 40–60 y/o women who fulfilled our inclusion criteria, 10 had incomplete reports of menopausal status and another 9 were excluded due to medical reasons including hysterectomy (3 cases), thyroid disease (3 cases), history of cancer (1 case) and medications (HRT, 2 cases). The remaining 43 women included 15 CNT, 14 PERI, and 14 MENO subjects, and were examined in this study.

Subjects’ characteristics are found in **Table 1**.

**Table 1:** Demographic and clinical characteristics by clinical group.

	Premenopause	Perimenopause	Postmenopause
N	15	14	14
Age, y, mean (SD), range	47(5), 40–55	50(6), 40–56	57(2), 52–60
Education, y, mean (SD)	16(2)	16(2)	16(2)
Family history of LOAD, % positive	66%	71%	79%
*APOE* ε4 carriers, % positive	46%	43%	36%
Ethnicity (%White)	80%	71%	86%
Subjective complaints (%)	80%	79%	100%
**Lab findings**			
Hypertension, % positive	13%	29%	14%
Body Mass Index (BMI)	25(6)	24(6)	24(4)
Hip to waist ratio [unitless]	1.0(0.4)	1.0(0.4)	1.1(0.1)
Systolic Blood pressure (mm/Hg)	113(18)	122(18)	118(9)
Diastolic blood pressure (mm/Hg)	70(13)	73(13)	69(8)
Fasting glucose (mg/dl)	66(24)	73(13)	76(14)
QUICKI score [unitless]	0.17(0.02)	0.18(0.02)	0.18(0.02)
Cholesterol (mg/dl)	189(63)	203(32)	229(33)^a^
HDL	66(27)	69(14)	82(22)
LDL	108(39)	118(28)	131(27)
Triglycerides (mg/dl)	72(44)	80(21)	79(35)
Homocysteine (micromol/l)	5.5(6.6)	9.1(5.7)	5.1(9.4)
Plasma vitamin B12 (ng/l)	419(268)	631(294)	657(507)
Plasma folate (ng/ml)	13(10)	15(4)	5(13) ^a^^,^^b^
**Neuropsychological tests**			
Mini Mental State Exam	29(2)	29(1)	29(1)
Digit symbol substitution	68(8)	67(12)	63(9)
Paragraph immediate recall	9(3)	7(2)	5(5)
Paragraph delayed recall	11(3)	9(3)	7(4)^a^
Paired associates Immediate recall	8(2)	6(3)	3(4)^a^
Paired associates delayed recall	8(3)	7(3)	5(5)
Designs	8(2)	8(2)	5(4)
Object naming	57(5)	58(2)	64(7)
WAIS-vocabulary	68(7)	65(8)	63(8)

Values are means (SD) unless otherwise specified. Abbreviations: CNT = asymptomatic perimenopause by age; PERI = symptomatic perimenopause by age; MENO = postmenopause.

^a^ MENO different from CNT

^b^ MENO different from PERI, p<0.05

As expected, the MENO group was older than CNT and PERI groups (p<0.05). Groups were otherwise comparable for demographical measures, frequency of a family history of AD, and distribution of APOE4 genotype (**Table 1**).

None of the participants were diabetic or met criteria for obesity as defined by a Body-Mass index (BMI)>30 kg/m^2^. The MENO group had a higher total cholesterol level compared to CNT, and reduced plasma folate compared to both CNT and PERI groups (p’s<0.03).

On neuropsychological testing, as compared to the CNT group, the MENO group showed lower memory scores on immediate and delayed recall (PRDI, PARD) tests (p’s<0.03), and a trend towards lower scores on delayed recall of paired associates (PRDD, p = 0.08) (**Table 1**). The MENO group also exhibited a trend towards lower PARD scores compared to the PERI group (p = 0.08).

### Mitochondrial COX activity

COX measures by clinical group are found in **Table 2**.

**Table 2:** Bioenergetic measures by transition state.

	Premenopause (n = 15)	Perimenopause (n = 14)	Postmenopause (n = 14)
**Mitochondrial measures**			
CS (nmol/min/mg)	142 (72)	157 (40)	145 (50)
COX (sec^-1^/mg)	41.5 (23.6)	36.4 (27.7)	30.9 (24.4)
Adjusted by age and CS	50.5 (15.9)	35.5 (16.6)^a^	21.5 (15.6)^b^
Adjusted by APOE	48.6 (16.0)	36.1 (16.7)^a^	23.8 (15.6)^a^
COX/CS (ratio, unitless)	0.30 (0.12)	0.23 (0.15)^a^	0.20 (0.15)^a^
Adjusted by age	0.34 (0.09)	0.24 (0.09)^a^	0.15 (0.10)^b^
Adjusted by age and APOE	0.32 (0.09)	0.24 (0.09)	0.16 (0.10)^a^
**CMRglc measures**			
AD-regions SUVR (unitless)	1.86 (0.12)	1.77 (0.18)	1.59 (0.09)^b^^,^^c^
Adjusted by age	1.91 (0.09)	1.76 (0.10)	1.54 (0.08) ^b^^,^^c^
Adjusted by age and APOE	1.88 (0.09)	1.78 (0.10)	1.56 (0.09) ^b^^,^^c^

Values are means (SD). Abbreviations: CS = citrate synthase, COX = cytochrome oxidase, SUVR = standardized FDG uptake value ratio to pons activity

^a^Different from CNT, p<0.05

^b^Different from CNT, p<0.01

^c^Different from PERI, p<0.01.

Results of these analyses, correcting for age and citrate synthase activity (CS), indicated that the total protein-referenced COX Vmax activity, as compared to CNT, was reduced by 30% in the PERI group (p = 0.04) and by 57% in the MENO group (p = 0.003). There was a trend towards reduced COX Vmax in MENO vs. PERI (39%, p = 0.09), yielding a gradient effect such as: CNT>PERI>MENO (p = 0.01).

When education and APOE were included as covariates in the model, there were no significant associations between these variables and COX activity, leaving group differences substantially unchanged (p<0.05, **Table 2**).

### FDG-PET glucose metabolism

**Fig 1:**
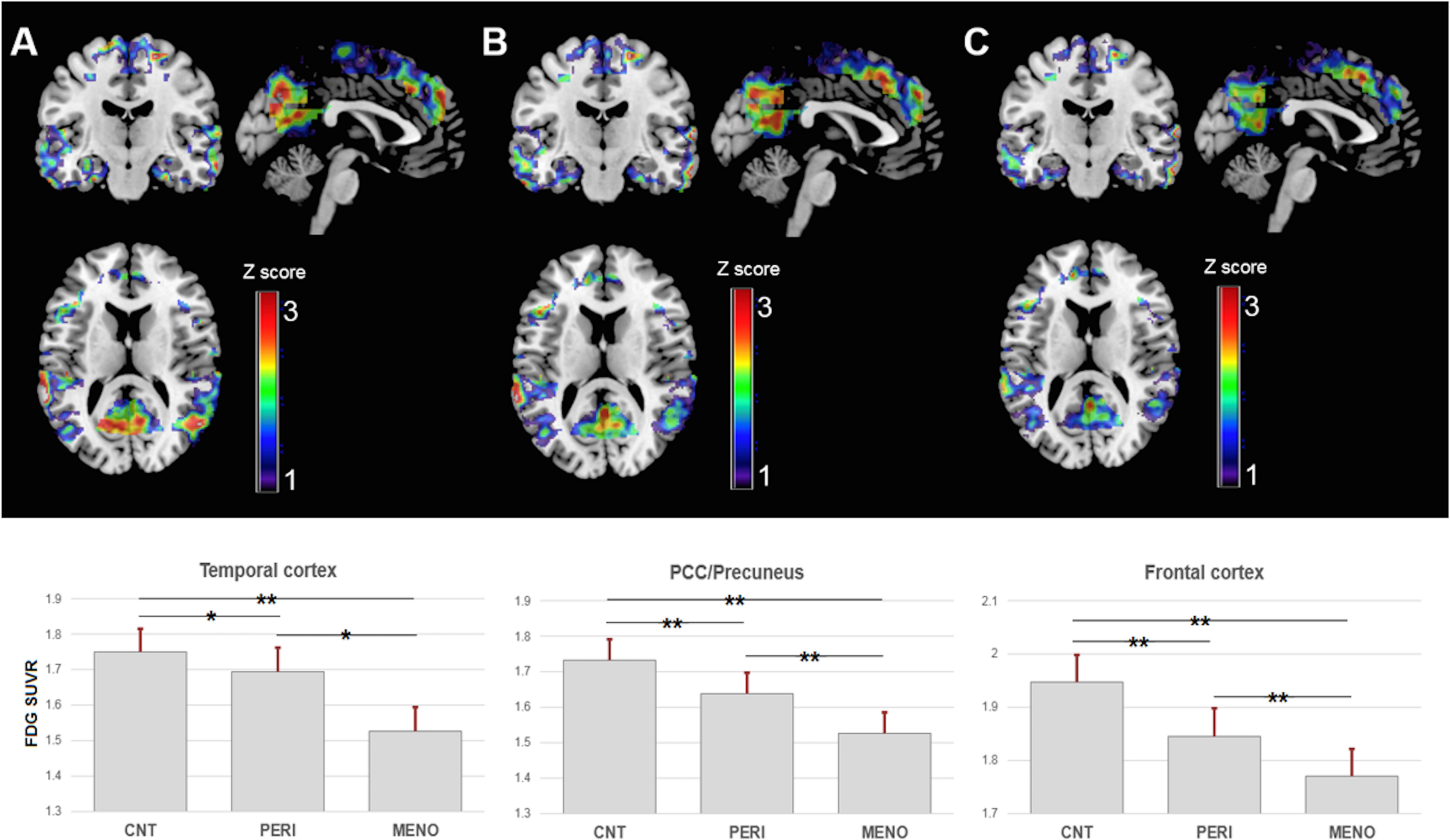
FDG-PET brain glucose metabolism as a function of endocrine aging. ***Top of figure***: Statistical parametric maps (SPMs) display reductions in ^18^F-fluoro-2-deoxyglucose (FDG) uptake in (A) postmenopausal (MENO) vs. premenopausal women (CNT); (B) perimenopausal (PERI) vs. premenopausal women; and (C) postmenopausal vs. perimenopauseal women. SPMs are represented on color-coded scales (1<z<3; where z>2 correspond to p<0.001) and displayed onto a standardized MRI. Corresponding coordinates and anatomical areas can be found in **S1 Table. *Bottom of figure***: CMRglc extracted from AD-regions by endocrine group. Values are pons-adjusted mean values, SEM; *p<0.01, **p<0.001. Abbreviations: CNT = premenopausal women; PERI = perimenopausal women; MENO = postmenopausal women, SUVR = standardized uptake value ratios (unitless).

Controlling for age and pons activity, CMRglc differences across groups were observed in AD-vulnerable posterior cingulate cortex/precuneus (PCC), frontal, parietal, medial and lateral temporal cortex, bilaterally (p<0.001, **Fig 1)**.

On post-hoc analysis, the MENO group exhibited CMRglc reductions in all the above regions as compared to CNT **(Fig 1** and **S1 Table).** The MENO group also showed CMRglc reductions in medial and lateral temporal cortex, bilaterally, and in PCC and middle frontal cortex of the left hemisphere as compared to the PERI group **(**p<0.001, **Fig 1** and **S1 Table).** The PERI group exhibited CMRglc reductions in temporal cortex, bilaterally, as well as in the inferior parietal cortex of the right hemisphere and PCC of the left hemisphere as compared to CNT (p<0.001, **Fig 1** and **S1 Table**). In CNT, no brain regions exhibited reduced CMRglc as compared to MENO or PERI groups.

As such, a gradient in CMRglc was evident in that: CNT>PERI>MENO (p<0.001, **Fig 1).** On average, age and pons-adjusted CMRglc in AD-regions was reduced by 19% in MENO vs, CNT, by 13% in MENO vs. PERI, and 8% in PERI vs. CNT (**Table 2**).

Results remained substantially unchanged including education and APOE as covariates (p’s<0.001).

### Associations between COX activity and CMRglc

**Fig 2:**
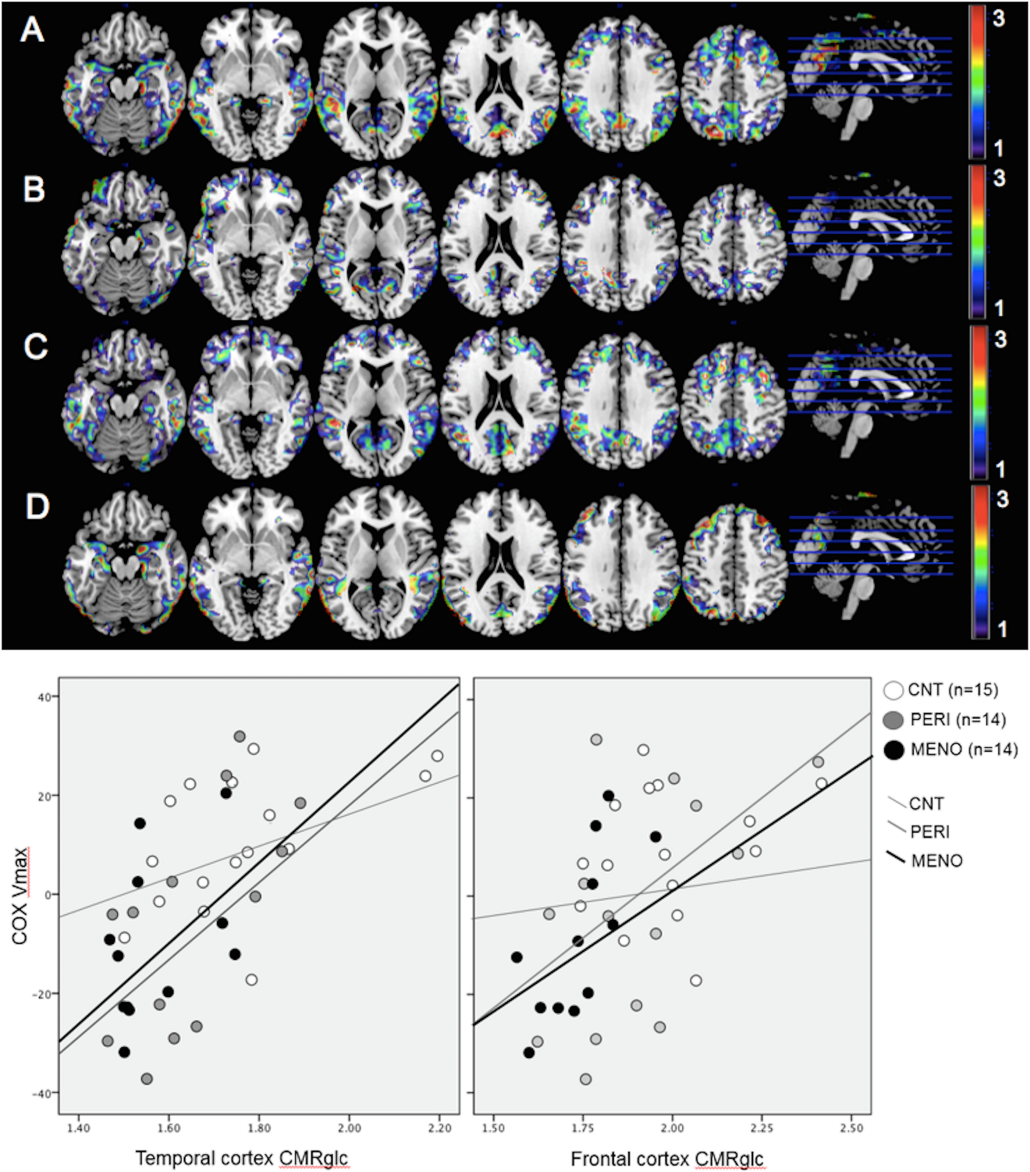
Associations between mitochondrial COX activity and FDG-PET brain glucose metabolism. ***Top of figure***: Statistical parametric maps (SPMs) display positive associations between CMRglc and COX in (A) all subjects, B) postmenopausal women, C) perimenopausal women, and D) premenopausal women. SPMs are represented on color-coded scales (1<z<3; where z>2 correspond to p<0.001) and displayed onto a standardized MRI. Corresponding coordinates and anatomical areas can be found in **S2 Table. *Bottom of figure***: Correlations between CMRglc in AD-regions and mitochondrial COX activity by endocrine group. CMRglc measures are age and pons-adjusted values; COX Vmax are age- and CS-adjusted residuals. All correlations p’s<0.001 except CNT p<0.05. Abbreviations: CNT = premenopausal women; PERI = perimenopausal women; MENO = postmenopausal women.

Correcting for the same confounds as above, across all participants, positive associations between COX activity and CMRglc were observed in PCC, frontal and temporal cortices (p<0.001, **Fig 2** and **S2 Table**).

The higher the COX activity, the higher the CMRglc in these regions, with FDG SUVR increasing by on average β_unstandardized_ = 0.004 units (SE = 0.001) for every unit increase in COX Vmax. No brain regions exhibited a negative correlation between COX and CMRglc.

Similar association patterns were observed within each group (**Fig 2** and **S2 Table).** Additionally, there was a significant interaction with endocrine group, with MENO and PERI groups exhibiting a steeper regression slope in frontal cortex CMRglc for COX compared to CNT (p_interaction_<0.05, **Fig 2).**

Metabolic markers were positively associated with memory scores (p<0.05). Specifically, both CMRglc and COX activity correlated with delayed memory scores (CMRglc: Pearson’s *r* = 0.26, β_unstd_ = 2.1, SE = 1.6, p = 0.05; COX Vmax: *r* = 0.31, β_unstd_ = 7.6, SE = 3.9, p = 0.04), and COX also correlated with immediate memory scores (*r* = 0.32, β_unstd_ = 9.3, SE = 4.2, p = 0.03).

### Prediction of menopausal status by biomarkers

To address whether bioenergetic measures are indicative of endocrine aging state, COX activity and or CMRglc were examined as predictors of endocrine state. Results are summarized in **Table 3**.

**Table 3:** Group separation as predicted by bioenergetic measures.

	% Sensitivity	% Specificity	% Accuracy	P	Relative Risk	95% C.I.
**Menopausal vs Premenopausal women**						
COX (*cutoff = 33*.*8* sec^-1^/mg)	64	87	79	0.008	5.2	1.4–19.8
CMRglc (*cutoff = 2*.*09 SUVR)*	100	93	96	<0.001	14.0	2.1–92.6
Combined CMRglc and COX	100	100	100	<0.001	28.9	1.9–442.3
**Perimenopausal vs Premenopausal women**						
COX (*cutoff = 36*.*02* sec^-1^/mg)	50	80	66	0.11	2.5	0.8–7.8
FDG SUVR (*cutoff = 1*.*77 SUVR)*	85	64	74	0.09	2.4	1.1–5.0
Combined CMRglc and COX	n.s.					
**Menopausal vs Perimenopausal women**						
COX (*cutoff = 33*.*7* sec^-1^/mg)	69	64	67	n.s.	1.9	0.9–4.2
FDG SUVR (*cutoff = 1*.*68 SUVR)*	92	54	72	0.002	2.0	1.1–3.7
Combined CMRglc and COX	n.s.					

*MENO vs CNT*. Both CMRglc (age and pons-adjusted) and COX activity (age and CS-adjusted) discriminated MENO from CNT, yielding 96% accuracy (Chi Sq_(1)_ = 28.5, relative risk, RR = 14, 95% C.I. 2.1–92.6, p<0.001) and 79% accuracy (Chi Sq_(1)_ = 7.1, RR = 5.2, 95% C.I. 1.4–19.8, p = 0.008), respectively. On stepwise forward analysis, adding COX to CMRglc measures in the model significantly increased the accuracy of CMRglc alone (p_increment_ = 0.01), for a combined 100% accuracy (ChiSq_(2)_ = 29.1, RR = 28.9, 95% C.I. 1.9–442.3, p<0.001).

*MENO vs PERI*. CMRglc discriminated MENO from PERI with 72% accuracy (Chi Sq_(1)_ = 20.5, relative risk, RR = 2.0, 95% C.I. 1.1–3.7, p = 0.002). COX activity was not a significant predictor of group membership, yielding 67% accuracy (p = 0.33, n.s.). Adding COX to CMRglc in the model did not increase the discrimination accuracy over CMRglc alone.

*PERI vs CNT*. Both CMRglc and COX showed non-significant trends towards discriminating PERI from CNT, yielding 74% and 67% accuracy, respectively (p≤0.11). Adding COX and CMRglc in the model did not increase the discrimination accuracy over either measure alone.

## Discussion

As compared to premenopausal women, perimenopausal and postmenopausal women exhibited brain hypometabolism in the same brain regions as clinical AD patients, as well as correlated reductions in mitochondrial COX activity. A gradient effect was observed so that bioenergetic abnormalities were most pronounced in postmenopausal, intermediate in perimenopausal, and lowest in premenopausal women. Results were independent of age, education, and APOE genotype.

Reduced FDG-PET CMRglc in posterior cingulate, parieto-temporal and frontal cortices is a well-established finding in patients with AD and with mild cognitive impairment (MCI), often an AD prodrome [33]. Further, PET studies in yet asymptomatic individuals demonstrate that CMRglc reductions in these key brain regions for AD precede and correlate with future cognitive decline and dementia [12–14]. Likewise, multiple studies report reduced COX activity in platelet mitochondria of MCI and AD patients, as well as of cognitively normal individuals at increased genetic risk of AD [25, 34].

Overall, our biomarker findings are indicative of a progressively increased risk of an AD endophenotype in women who undergo the perimenopause to menopause transition, and suggest that endocrine aging outweighs the effects of chronological aging in the female’s brain several years, if not decades, before possible clinical symptoms emerge.

These findings are particularly relevant given our current understanding of AD as a progressive disorder characterized by an extended preclinical phase during which the disease is underway but hasn’t led to recognizable clinical or cognitive symptoms [5]. Given the known relationship between altered brain bioenergetics and onset of AD symptoms, our data indicate that sex-specific hormonal stages such as the perimenopause and postmenopause may at least in part account for the increased AD risk observed in women.

The question of where bioenergetic dysfunction sits in the pathogenesis of late-onset AD is an area of active debate. According to the amyloid cascade hypothesis, oxidative stress arises downstream of Aß dysmetabolism, particularly in the rare, early-onset familial AD cases that associate with autosomal dominant genetic mutations[35].

It is possible that our findings of altered bioenergetics in aging women may depend on ongoing Aß deposition. Cell culture experiments and studies of transgenic mice expressing mutant human amyloid-precursor protein (APP) indeed show COX inhibition or altered COX gene expression[20]. Brain imaging studies indicate that amyloid deposition predates hypometabolism in early-onset familial AD cases [17, 36], although results are less consistent in the most common late-onset form of AD [37].

Alternatively, reduced metabolic activity in perimenopausal and postmenopausal women could potentially arise as a consequence of a bioenergetic crisis in brain during the perimenopause [4]. Preclinical studies indicate that during perimenopause, when estrogen in brain plummets, the systems required for estrogen activation of CMRglc and suppression of the ketogenic pathway are disassembled [38]. Following perimenopause, a response to decline in CMRglc is induction of an adaptive starvation reaction to increase fatty acid metabolism for the generation and utilization of ketone bodies by mitochondria as an alternative fuel [4, 38–41]. Hypometabolism, reduced mitochondrial function and subsequent oxidative damage are known to promote accumulation of Aß pathology and neuronal dysfunction [42], therefore increasing risk of developing AD later in life.

Our biomarker findings in humans support the mechanistic pathway from animal studies demonstrating that perimenopausal and postmenopausal stages are associated with an energetically compromised brain in women, as reflected in brain hypometabolism and associated reduction in mitochondrial function. Blood platelets are a peripheral, non-degenerating tissue that should not be affected by CNS pathology [11, 43, 44]. This suggests that decreased bioenergetics during menopause is not simply a secondary consequence of neurodegeneration but may instead represent a systemic deficit at least in some women. As mitochondria are essential sites for steroid hormone biosynthesis[45], more studies are needed to address the possibility that mitochondrial dysfunction is an upstream event in the menopausal estrogen drop leading to altered brain bioenergetics. As amyloid imaging was not available for many of our subjects, future studies are needed to determine whether amyloid pathology influences the associations between neuroendocrine transitions, brain hypometabolism, declines in COX activity, and cognition.

Since this is the first study demonstrating perimenopausal and postmenopausal effects on bioenergetic markers, several questions remain to be answered.

First, our cross-sectional results do not allow for determination of causality or temporal relationships between biomarkers and clinical status, nor do they offer information on future amyloid deposition and conversion to AD. In our study, metabolic markers were associated with memory performance, especially on delayed recall tests known to be sensitive to estrogen declines in women [46], though the associations were modest. Other studies with larger samples and longitudinal follow-ups are warranted to determine whether the bioenergetic abnormalities observed in perimenopausal and postmenopausal women are predictive of cognitive decline and dementia.

Although MRS imaging can be used to measure mitochondrial activity in brain, there are no techniques that enable examination of COX activity, specifically. As such, it remains to be established whether the degree of the observed platelet mitochondria COX activity reductions correlates with a corresponding reduction in brain mitochondria COX activity. Post-mortem studies showed significant correlations between brain histology and *in vivo* blood platelet COX measurements in AD and control subjects[47]. We offer that the platelet COX defect would underestimate the brain COX defect, as neurons are more easily affected by oxidative damage than other tissues and their long life facilitates an accumulation of somatic mtDNA modifications [11]. If this assumption is correct, then persons with low platelet mitochondria COX activity would have less COX reserve in brain, and would therefore be more likely over time to reach a point of bioenergetic compromise. Present findings of positive correlations between peripheral COX Vmax and CMRglc in brain regions known to be vulnerable to oxidative stress and AD support this hypothesis. Other measures of mitochondrial function, such as metabolomic studies in blood and/or CSF, are of interest as they would allow measurement of lactate, pyruvate, and related metabolites in the perimenopause to menopause transition. Likewise, novel PET tracers for mitochondrial activity are currently being tested in animals, including primates, for possible application in humans[48].

Present findings were independent of APOE genotype, a well-known genetic risk factor for late-onset AD [49] which associates with increased risk of AD in women more than in men [1, 50]. Future studies with larger samples are needed to specifically examine the interactions between endocrine aging and APOE4 status on bioenergetics as well as other AD-biomarkers.

Our determination of menopausal state in the absence of hormonal confirmation is vulnerable to error. Our determination of reproductive stage was based on self-report, clinical judgment, and established diagnostic criteria known to have good agreement with clinical and lab findings [24], which reduce potential for misclassification. While we consider it more likely that the changes in menstrual cycle frequency reported by our participants reflect their actual menopausal status, because of the synchronous timing of medical assessments and brain imaging exams, our menopausal group may have included subjects still in perimenopause. Likewise, our asymptomatic controls may have included subjects undergoing perimenopausal changes. This would, however, conservatively reduce power in detecting differences between groups. Our findings of gradual increases in biomarker abnormalities in perimenopausal and postmenopausal subjects vs. premenopausal controls are consistent with preclinical and neuropsychological indicators of change in cognitive function [51–53], and provide support that that our group assignment criteria were likely correct. Finally, age at surgical menopause was found to influence cognitive decline and Alzheimer’s amyloid pathology in a large cohort of older women[54]. We did not include any participants with surgically-induced menopause. Prospective studies are warranted to provide accurate determination of age at menopause in naturally-occurring menopausal women, and test its associations with FDG and COX activity.

None of the postmenopausal women included in this study were on hormonal replacement therapy (HRT). Clinical trials have shown that HRT is effective at preserving CMRglc in AD-regions, especially if initiated prior to menopause [55, 56] whereas it can be deleterious when initiated after menopause [57, 58] or in Type 2 diabetic women [55, 59, 60]. Our biomarker results support further investigation of the potential efficacy of estrogen-based therapies in preventing decline in brain bioenergetic capacity in women at the perimenopausal stage.

We caution that present results were found in a small cohort of carefully screened patients, mostly representative of the New York area. Results may differ in populations including women with higher BMI, lower education levels, or increased risk for metabolic syndrome at earlier age. Replication of these preliminary findings in community-based populations with more diversified socio-economic and medical status, as well as with other biomarkers of AD, is warranted and clinical application is not yet justified.

## Supporting information

S1 Table Brain regions showing significant differences in FDG uptake across female groups. *Coordinates (x, y, z) from Talairach and Tournoux. ^†^Z values at the peak of maximum significance at p<0.001, corrected for the search volume. Only contrasts yielding significant results are reported. FDG measures are age-adjusted cortical-to-pons standardized uptake volume ratios.
(DOCX)

S2 Table Brain regions showing significant positive associations between mitochondrial COX activity and FDG uptake across female groups. *Coordinates (x, y, z) from Talairach and Tournoux. ^†^Z values at the peak of maximum significance at p<0.001, corrected for the search volume. Only contrasts yielding significant results are reported. FDG measures are age-adjusted cortical-to-pons standardized uptake volume ratios.
(DOCX)
