# Correction: Perimenopause and emergence of an Alzheimer's bioenergetic phenotype in brain and periphery

**DOI:** 10.1371/journal.pone.0193314

**Published:** 2018-02-15

**Authors:** Lisa Mosconi, Valentina Berti, Crystal Quinn, Pauline McHugh, Gabriella Petrongolo, Ricardo S. Osorio, Christopher Connaughty, Alberto Pupi, Shankar Vallabhajosula, Richard S. Isaacson, Mony J. de Leon, Russell H. Swerdlow, Roberta Diaz Brinton

The third author’s name is incorrect. The correct name is: Crystal Quinn. The correct citation is: Mosconi L, Berti V, Quinn C, McHugh P, Petrongolo G, Osorio RS, et al. (2017) Perimenopause and emergence of an Alzheimer’s bioenergetic phenotype in brain and periphery. PLoS ONE 12(10): e0185926. https://doi.org/10.1371/journal.pone.0185926
