# Supplementary material for: Perimenopause and emergence of an Alzheimer’s bioenergetic phenotype in brain and periphery
Source: PLoS One. 2017 Oct 10;12(10):e0185926. doi: 10.1371/journal.pone.0185926 (PMC5634623; doi:10.1371/journal.pone.0185926)
Supplement: S1 Table — *Coordinates (x, y, z) from Talairach and Tournoux. †Z values at the peak of maximum significance at p<0.001, corrected for the search volume. Only contrasts yielding significant results are reported. FDG measures are age-adjusted cortical-to-pons standardized uptake volume ratios. (DOCX) [file pone.0185926.s001.docx]

**S1 Table.** Brain regions showing significant differences in FDG uptake across female groups.

| **Cluster extent** | **T** | **Z^†^** | **Coordinates**** | | | **Hemisphere** | **Anatomical area** | **Brodmann area** |
| --- | --- | --- | --- | --- | --- | --- | --- | --- |
|  |  |  | X | Y | Z |  |  |  |
| **Regional Hypometabolism in Menopausal vs Premenopausal Women** | | | | | | | | |
| 81 | 5.44 | 4.60 | 65 | -15 | -17 | Right | Inferior Temporal Gyrus | BA 21 |
| 81 | 5.44 | 4.60 | 65 | -15 | -17 | Right | Inferior Temporal Gyrus | BA 21 |
|  | 3.59 | 3.29 | 62 | -9 | -11 | Right | Middle Temporal Gyrus | BA 21 |
| 302 | 4.76 | 4.15 | -63 | -39 | 0 | Left | Middle Temporal Gyrus | BA 21 |
|  | 4.30 | 3.83 | -63 | -29 | 11 | Left | Superior Temporal Gyrus | BA 42 |
|  | 3.80 | 3.45 | -62 | -38 | 11 | Left | Superior Temporal Gyrus | BA 22 |
| 218 | 4.11 | 3.69 | -34 | -47 | -9 | Left | Fusiform Gyrus | BA 37 |
| 66 | 4.02 | 3.62 | -21 | -70 | 48 | Left | Precuneus | BA 7 |
| 44 | 3.89 | 3.52 | -59 | -8 | -24 | Left | Fusiform Gyrus | BA 20 |
|  | 3.51 | 3.22 | -59 | -17 | -24 | Left | Fusiform Gyrus | BA 20 |
| 45 | 3.87 | 3.50 | 67 | -21 | -7 | Right | Middle Temporal Gyrus | BA 21 |
|  | 3.59 | 3.29 | 65 | -27 | -13 | Right | Middle Temporal Gyrus | BA 21 |
|  | 3.52 | 3.23 | 63 | -26 | 22 | Right | Inferior parietal lobule | BA 40 |
| 25 | 3.77 | 3.43 | -61 | -27 | 35 | Left | Inferior Parietal Lobule | BA 40 |
| 70 | 3.68 | 3.36 | -30 | -54 | 50 | Left | Superior Parietal Lobule | BA 7 |
| 31 | 3.61 | 3.31 | 3 | 50 | 34 | Right | Medial Frontal Gyrus | BA 9 |
| 25 | 3.60 | 3.30 | -44 | 7 | 25 | Left | Inferior Frontal Gyrus | BA 9 |
| 25 | 3.35 | 3.10 | -24 | -74 | 38 | Left | Precuneus | BA 7 |
| **Regional Hypometabolism in Menopausal vs Perimenopausal Women** | | | | | | | | |
| 586 | 4.57 | 4.02 | -62 | -43 | 13 | Left | Superior Temporal Gyrus | BA 22 |
|  | 4.22 | 3.77 | -63 | -41 | 2 | Left | Middle Temporal Gyrus | BA 21 |
|  | 4.21 | 3.76 | -61 | -27 | 35 | Left | Inferior Parietal Lobule | BA 40 |
| 129 | 4.20 | 3.75 | 65 | -15 | -14 | Right | Middle Temporal Gyrus | BA 21 |
|  | 3.79 | 3.44 | 67 | -22 | -8 | Right | Middle Temporal Gyrus | BA 21 |
| 182 | 4.16 | 3.72 | -30 | -54 | 50 | Left | Superior Parietal Lobule | BA 7 |
|  | 3.30 | 36 | -36 | -48 | 41 | Left | Inferior Parietal Lobule | BA 40 |
| 33 | 4.14 | 3.71 | -61 | -40 | 38 | Left | Inferior Parietal Lobule | BA 40 |
| 71 | 4.07 | 3.66 | 20 | -29 | -6 | Right | Parahippocampal Gyrus | BA 28 |
| 188 | 4.02 | 3.62 | -36 | -47 | -9 | Left | Fusiform Gyrus | BA 37 |
|  | 3.36 | 3.10 | -30 | -38 | -6 | Left | Parahippocampal Gyrus | BA 37 |
| 27 | 3.96 | 3.57 | -59 | -10 | -24 | Left | Fusiform Gyrus | BA 20 |
| 84 | 3.85 | 3.49 | -45 | -57 | 29 | Left | Superior Temporal Gyrus | BA 39 |
|  | 3.59 | 3.29 | -34 | -55 | 26 | Left | Middle Temporal Gyrus | BA 39 |
| 70 | 3.75 | 3.42 | -15 | -61 | 54 | Left | Superior Parietal Lobule | BA 7 |
| 23 | 3.58 | 3.28 | -20 | -7 | 61 | Left | Middle Frontal Gyrus | BA 6 |
| 58 | 3.42 | 3.15 | -18 | -71 | 48 | Left | Precuneus | BA 7 |
|  | 3.26 | 3.02 | -12 | -75 | 42 | Left | Precuneus | BA 7 |
| 21 | 3.41 | 3.15 | -61 | -47 | -9 | Left | Middle Temporal Gyrus | BA 37 |
| **Regional Hypometabolism in Perimenopausal vs Premenopausal Women** | | | | | | | | |
| 35 | 5.30 | 4.51 | 65 | -15 | -17 | Right | Inferior Temporal Gyrus | BA 21 |
| 119 | 4.34 | 3.86 | -63 | -39 | 0 | Left | Middle Temporal Gyrus | BA 21 |
|  | 3.93 | 3.55 | -63 | -29 | 11 | Left | Superior Temporal Gyrus | BA 42 |
|  | 3.37 | 3.11 | -62 | -37 | 10 | Left | Superior Temporal Gyrus | BA 22 |
| 101 | 3.74 | 3.41 | -33 | -47 | -8 | Left | Fusiform Gyrus | BA 37 |
| 25 | 3.69 | 3.37 | -21 | -70 | 48 | Left | Precuneus | BA 7 |
| 32 | 3.54 | 3.25 | 67 | -23 | 27 | Right | Inferior Parietal Lobule | BA 40 |

*Coordinates (x, y, z) from Talairach and Tournoux. ^†^Z values at the peak of maximum significance at p<0.001, corrected for the search volume. Only contrasts yielding significant results are reported.

FDG measures are age-adjusted cortical-to-pons standardized uptake volume ratios.
