# Supplementary material for: Perimenopause and emergence of an Alzheimer’s bioenergetic phenotype in brain and periphery
Source: PLoS One. 2017 Oct 10;12(10):e0185926. doi: 10.1371/journal.pone.0185926 (PMC5634623; doi:10.1371/journal.pone.0185926)
Supplement: S2 Table — *Coordinates (x, y, z) from Talairach and Tournoux. †Z values at the peak of maximum significance at p<0.001, corrected for the search volume. Only contrasts yielding significant results are reported. FDG measures are age-adjusted cortical-to-pons standardized uptake volume ratios. (DOCX) [file pone.0185926.s002.docx]

**S2 Table.** Brain regions showing significant positive associations between mitochondrial COX activity and FDG uptake across female groups.

| **Cluster extent** | **T** | **Z^†^** | **Coordinates*** | | | **Hemisphere** | **Anatomical area** | **Brodmann area** |
| --- | --- | --- | --- | --- | --- | --- | --- | --- |
|  |  |  | X | Y | Z |  |  |  |
| **All subjects** | | | | | | | | |
| 124 | 4.54 | 4.01 | 27 | 5 | 55 | Right | Superior Frontal Gyrus | BA 6 |
| 66 | 4.19 | 3.76 | 56 | -59 | 0 | Right | Middle Temporal Gyrus | BA 37 |
|  | 3.36 | 3.11 | 59 | -56 | -7 | Right | Inferior Temporal Gyrus | BA 37 |
| 76 | 3.99 | 3.61 | 8 | 6 | 67 | Right | Superior Frontal Gyrus | BA 6 |
| 42 | 3.92 | 3.56 | -39 | -53 | 16 | Left | Superior Temporal Gyrus | BA 22 |
| 35 | 3.74 | 3.42 | 22 | -13 | 59 | Right | Middle Frontal Gyrus | BA 6 |
| 23 | 3.64 | 3.34 | -62 | -42 | -13 | Left | Middle Temporal Gyrus | BA 20 |
| 42 | 3.61 | 3.31 | 10 | -70 | 23 | Right | Precuneus | BA 31 |
| 39 | 3.60 | 3.30 | -9 | -3 | 70 | Left | Superior Frontal Gyrus | BA 6 |
| 22 | 3.58 | 3.29 | -40 | 2 | -12 | Left | Superior Temporal Gyrus | BA 38 |
| 83 | 3.57 | 3.28 | -30 | -66 | 43 | Left | Precuneus | BA 19 |
|  | 3.29 | 3.06 | -20 | -66 | 48 | Left | Precuneus | BA 7 |
| 44 | 3.54 | 3.26 | 45 | -50 | 14 | Right | Superior Temporal Gyrus | BA 39 |
| **Premenopausal women** | | | | | | | | |
| 54 | 6.68 | 4.14 | -22 | -58 | 31 | Left | Precuneus | BA 7 |
| 35 | 6.44 | 4.07 | -21 | 36 | -24 | Left | Inferior Frontal Gyrus | BA 11 |
| 23 | 6.27 | 4.01 | -51 | 3 | -33 | Left | Middle Temporal Gyrus | BA 21 |
| 58 | 5.73 | 3.82 | -16 | -48 | 30 | Left | Precuneus | BA 31 |
| 28 | 5.29 | 3.66 | -10 | -15 | 49 | Left | Medial Frontal Gyrus | BA 6 |
| 20 | 4.57 | 3.35 | -57 | -8 | -30 | Left | Inferior Temporal Gyrus | BA 20 |
| **Perimenopausal women** | | | | | | | | |
| 25 | 6.17 | 3.88 | 8 | -60 | 33 | Right | Precuneus | BA 7 |
| 30 | 5.46 | 3.64 | -51 | -47 | 37 | Left | Inferior Parietal Lobule | BA 40 |
| **Postmenopausal women** | | | | | | | | |
| 45 | 6.79 | 3.95 | -55 | 6 | 28 | Left | Inferior Frontal Gyrus | BA 9 |
| 22 | 6.01 | 3.72 | -22 | -95 | -12 | Left | Fusiform Gyrus | BA 18 |
| 21 | 5.67 | 3.61 | -63 | -50 | 3 | Left | Middle Temporal Gyrus | BA 21 |
| 59 | 5.64 | 3.60 | 22 | 2 | 67 | Right | Superior Frontal Gyrus | BA 6 |
|  | 4.38 | 3.13 | 14 | 6 | 67 | Right | Superior Frontal Gyrus | BA 6 |
| 36 | 5.10 | 3.41 | -65 | -35 | 5 | Left | Middle Temporal Gyrus | BA 22 |
| 66 | 5.00 | 3.37 | 40 | 0 | 58 | Right | Middle Frontal Gyrus | BA 6 |
| 33 | 4.93 | 3.35 | 14 | -8 | 72 | Right | Superior Frontal Gyrus | BA 6 |
|  | 3.99 | 2.95 | 14 | 0 | 67 | Right | Superior Frontal Gyrus | BA 6 |
| 51 | 4.72 | 3.27 | -38 | -3 | 58 | Left | Middle Frontal Gyrus | BA 6 |
|  | 4.66 | 3.24 | -33 | -9 | 62 | Left | Precentral Gyrus | BA 6 |
|  | 4.13 | 3.02 | -38 | 6 | 56 | Left | Middle Frontal Gyrus | BA 6 |

*Coordinates (x, y, z) from Talairach and Tournoux. ^†^Z values at the peak of maximum significance at p<0.001, corrected for the search volume. Only contrasts yielding significant results are reported. FDG measures are age-adjusted cortical-to-pons standardized uptake volume ratios.
